# Sensing of micropillars by osteoblasts involves complex intracellular signaling

**DOI:** 10.1007/s10856-017-5982-8

**Published:** 2017-09-27

**Authors:** Caroline Moerke, Petra Mueller, J. Barbara Nebe

**Affiliations:** Department of Cell Biology, University Medical Center Rostock, Schillingallee 69, 18057 Rostock, Germany

## Abstract

**Abstract:**

Topographical material surface features are sensed by cells and provoke a large range of cellular responses. We recognized earlier, that at micropillar topographies in the range of 5 µm, the osteoblasts attempt to phagocytize the pillars resulted in increased energy requirements and reduced osteoblast marker expression, e.g., collagen type I and osteocalcin. However, the precise cellular signaling transducing the topographic information into the cell and evoking phagocytic processes remained unknown. Here, we could show that the RhoA/ROCK signaling is involved in the transduction of the topography-mediated cellular reactions. After inhibition of ROCK-2 with Y27632 for 24 h, no caveolae-mediated micropillar assembly of the cell membrane domain component caveolin-1 (Cav-1) was found. ROCK inhibition was also able to attenuate the pillar-induced decrease in β-actin. Interestingly, phosphatidylinositol 3-kinase (PI3K) inhibition with LY294002 for 24 h did not influence the Cav-1 clustering on micropillars. Our results illustrate the importance of the integrin down-stream signaling of RhoA/ROCK in the recognition of and adaption to surface microtopographies by osteoblasts and extend our understanding about the complex mechanism of action inside the cells.

**Graphical abstract:**

## Introduction

In cell-biomaterial interaction, knowledge of the dependence of cell behavior on topographical features is relevant for the design of implant surfaces. Cells are able to recognize surface topographies of micron as well as nanometer size and adapt their cellular behavior by sensing topographies down to 10 nm with their filopodia [1, 2]. Micron-scale topography has been reported to induce changes in cell adhesion, morphology, motility and gene expression [1]. Despite intensive research, the principles of cellular responses to surface topography are not completely understood. Because many variables influence cellular interactions to surface structures, e.g., wettability, surface charges, feature curvature or stiffness, general cell behavior principles for nano- and microtopographies could not be established [3]. The first cellular attachment, adhesion and spreading will influence the cells’ capacity to proliferate and to differentiate in contact with the material [4]. This complex process includes various biological components such as the cell adhesion receptors, the integrins, and the actin cytoskeleton, which are connected via adapter proteins in functional units called focal adhesions. These adaptor proteins are co-localized with kinases and phosphatases, e.g., focal adhesion kinase (FAK) and Src, transducing the signals to the nucleus for regulation of gene expression [5]. This integrin-mediated sensing of the extracellular matrix (ECM) composition as well as topography is called “outside-in” signaling [6]. Thus, integrins function as mechanotransducers of extracellular signals that determine subsequent cellular processes such as cell adhesion, spreading, migration and consequently also cell survival, proliferation and differentiation [7, 8].

Integrin-dependent functions can be altered by cellular morphology changes and can modulate the integrin-activated signaling mediators. Rho-family GTPases and their downstream kinase Rho-associated kinase (ROCK) relay integrin-derived signals; they also organize the actin cytoskeleton. Therefore, it is suggested that they integrate cell shape and function [9]. Recently, we discovered an attempted caveolae-mediated phagocytosis of surface-fixed micropillars by human MG-63 osteoblastic cells [8, 10]. This attempt to phagocytize the cubic elevations of the Ti surface results in altered actin cytoskeleton organization [8, 11] and higher energy metabolism, leading to increased generation of intracellular reactive oxygen species (ROS). Finally, this behavior caused a decreased expression of osteoblast differentiation proteins such as collagen, fibronectin, osteocalcin and alkaline phosphatase [8]. The regulation of the phagocytic process involves signaling pathways including extracellular signal-regulated kinase 1/2 (ERK1/2) and actin cytoskeleton organization. These are implicated in exterior mechanical and force regulation, besides their function in cell growth, differentiation and stress response [12–14]. Phagocytosis is a process involving cell mobility or directed migration around the internalized cargo. The phosphatidylinositol 3-kinase (PI3K) is crucial for phagocytic engulfment, but also for cell adhesion and migration [15]. This highlights the tightly linked signaling cascades between phagocytosis, cell adhesion and migration [16].

In this study, we examined the cell signaling in human MG-63 osteoblasts depending on the underlying micropillar topography and the topography-triggered cell changes. The artificial micropillar topography has the advantage of constant repetitive dimensions and facilitates the specific analysis of topography-induced cellular processes; it also highlighted the importance of the cell-material contact area for the osteoblasts in maintaining their characteristic osteoblast function [8] and showed how this contact can manipulate cell reactions.

## Materials and methods

### Microtextured titanium surfaces

Periodically microtextured samples (size 1 cm²) with regular cubic pillar geometry on the surface having a dimension of 5 × 5 × 5 µm in width x length x height and 5 µm in spacing (P-5 × 5) were used. Unstructured, planar silicon wafers (Ref) were employed as controls. The samples were fabricated by deep reactive-ion etching (DRIE) (Center for Microtechnologies ZFM, University of Technology Chemnitz, Germany) on silicon wafers and coated with an additional 100 nm titanium (Ti) layer, as reported before [8, 10].

### Osteoblast cell culture

The human osteoblast-like cells MG-63 (American Type Culture Collection ATCC®, CRL-1427) were cultivated in Dulbecco’s modified eagle medium (DMEM, Life Technologies GmbH, Darmstadt, Germany) with 10% fetal calf serum (FCS) (Biochrom FCS Superior, Merck KGaA, Darmstadt, Germany), as was reported before [8, 10]. For phosphatidylinositol 3-kinase (PI3K) inhibition the cells were treated with 10 µM LY294002 (Cell Signaling Technology Inc., Danvers, MA, USA), and for ROCK inhibition with 20 µM Y27632 (Cell Signaling Technology Inc.) during the 24 h culture time. The inhibitory substances were diluted in dimethylsulfoxide (DMSO, Merck KGAA, Darmstadt, Germany) to a stock solution of 10 mM. The control experiments were also carried out with the appropriate amount of the vehicle DMSO.

### Western-blotting

Immunoblots were performed from total lysates of MG-63 cells which were cultivated on the Ti arrays. The BioPlex cell lysis kit (Bio-Rad Laboratories GmbH, Munich, Germany) was used. Protein quantification was performed using the Bradford method (Bio-Rad Laboratories GmbH). Total cellular protein was separated by SDS-PAGE (Bio-Rad Laboratories GmbH) and afterwards transblotted to polyvinylidine fluoride (PVDF) membranes (Roche Diagnostics GmbH, Mannheim, Germany). Analyses were done with the following antibodies: β-actin mouse monoclonal (Santa Cruz Biotechnologies Inc., Dallas, TX, USA), caveolin-1 rabbit polyclonal (New England Biolabs GmbH; Frankfurt/Main, Germany), Tyr14 phosphorylated caveolin-1 rabbit monoclonal (BD Biosciences, Franklin Lakes, NJ, USA; 1:500), FAK mouse polyclonal (BD Transduction), ROCK-2 pY256 rabbit monoclonal (Rockland Inc. Limerick, PA, USA), ROCK-2 rabbit polyclonal (Santa Cruz Biotechnologies Inc.), RPLP0 (60S acidic ribosomal protein P0) mouse monoclonal (LifeSpan BioScience Inc.,Seattle, WA, USA), and Src mouse monoclonal (New England Biolabs GmbH). The membranes were incubated with the appropriate primary antibody over-night at 4°C followed by a horseradish peroxidase (HRP)-conjugated secondary antibody (New England Biolabs GmbH). Primary antibody binding was detected by using a fast chemiluminescent substrate for HRP (Femto Dura West Signal, Thermo Scientific). For each protein detected, at least four independent experiments were performed. Immunoblotting analyses were carried out with ImageLab-ChemiDoc-MP (Bio-Rad Laboratories GmbH) and the densitometric analysis with ImageJ (Wayne Rasband, National Institute of Health). The 60S acidic ribosomal protein P0 (RPLP0) as well as the stain-free gel with total protein loading were used as an endogenous control. For the evaluation of the phosphorylation states, the normalized phosphorylated protein was calculated relative to the normalized unphosphorylated, total protein.

### Luminex assay

Total lysates of MG-63 cells were prepared with the BioPlex cell lysis kit (Bio-Rad Laboratories GmbH) and protein quantification was performed using the Bradford method (Bio-Rad Laboratories GmbH), just as for the immunoblots. For the luminex assay measurements, the following analytes and kits were used: for the focal adhesion kinase (FAK) (pY397) and FAK (pY861) the MILLIPLEX MAP Kit (Merck KGAA, Darmstadt, Germany), and for Src (pY419) the MILLIPLEX MAP Human Src Family Kinase Kit (Merck KGAA). Protocols were performed according to the manufacturer’s instruction in a 96-well plate with 25 µl of the protein lysates for cellular protein quantification. Protein quantification was done using the Bio-Plex-System (Bio-Rad Laboratories GmbH) and the Bio-Plex Manager™ 4.1.1 software (Bio-Rad Laboratories GmbH), measured in mean fluorescence intensity (MFI). The MFI values were normalized using protein lysate concentrations measured according to the Bradford method. For the evaluation of the phosphorylation states, the normalized phosphorylated protein was calculated relative to the normalized unphosphorylated, total protein.

### Immunofluorescence

Immunofluorescence staining was performed as reported before [8, 10]. Briefly, cells were fixed with 4% paraformaldehyde (10 min at room temperature, RT) (Sigma-Aldrich), washed with PBS, permeabilized with 0.1% Triton X-100 (10 min, RT) (Merck KGaA), and blocked with 2% bovine serum albumin (BSA) (Sigma-Aldrich) in PBS (30 min, RT). For actin filament staining, the cells were incubated with phalloidin coupled with tetramethyl-rhodamine (TRITC) (5 µg/ml in PBS, Sigma-Aldrich). The following antibodies were used: caveolin-1 rabbit polyclonal (Cav-1, New England Biolabs GmbH), PIP2 anti-phosphatidylinositols mouse monoclonal antibody (Abcam, Cambridge, MA, USA) and the secondary antibodies anti-mouse-IgG-AF488 as well as anti-rabbit-IgG-488(Life Technologies). The samples were embedded with a fluoroshield mounting medium (Sigma-Aldrich). The experiments were repeated three times.

### Confocal scanning microscopy and cell area quantification

Image acquisition was done on an inverted confocal laser scanning microscope LSM 780 (Carl Zeiss AG) using the ZEISS oil immersion objective (C-Apochromat63) and the ZEN 2011 (black version) software (Carl Zeiss AG). All images were displayed as three-dimensional (3D) z-stacks (13 stacks with an interval of 1 µm; frame sizes of 1250 × 1015 pixels). Z-stacking was used to generate a 3D representation to understand the overall cell structures. Thus, a false interpretation due to different observation levels (confocal principle) could be avoided. The cell areas for *n* = 50 cells per sample out of *n* = 2 experiments with ImageJ software (Wayne Rasband, National Institute of Health) using the LSM images from the actin cytoskeleton staining.

### Statistical analyses

Statistical analyses were carried out with GraphPad Prism5 software (GraphPad Software Inc.). Results are presented in box plots with medians, quartiles and an interquartile range (IQR) ± 1.5 x IQR. Data analyses were performed using the Mann–Whitney U test. *P*-values <0.05 were considered to indicate significant differences.

## Results

### Signaling proteins

**Fig. 1:**
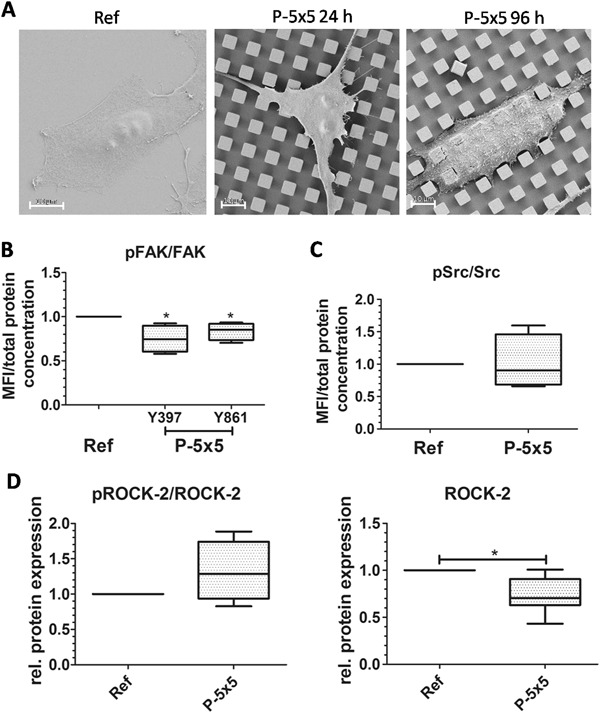
**a** Scanning electron microscopy images of the Ti planar reference (Ref) and the 5 × 5 × 5 µm micropillars (P-5 × 5) with human MG-63 osteoblasts after 24 and 96 h (FESEM Merlin VP, Carl Zeiss, bars 10 µm). **b** MG-63 cells on micropillars for 24 h: quantification of phosphorylated focal adhesion kinase (FAK) at Y397 and Y861 via luminex assay. **c** MG-63 cells on micropillars for 24 h: quantification of phosphorylated Src at Y419 via luminex assay. (For **b**, **c**: Ref values normalized on 1, *n* = 4, Mann-Whitney U test, *P < 0.05, *MFI* mean fluorescence intensity). **d** MG-63 cells on micropillars for 24 h: Phosphorylated ROCK-2 at Y256 and ROCK-2 via Western-blotting. (Ref values normalized on 1, *n* = 8, Mann–Whitney U test, *: *P* < 0.05)

The overall cell interaction with the microtopography is illustrated in Fig. 1. After 24 h the osteoblasts spread on top of the micropillars. They only have the pillar plateaus as adhesion sites thus having less adhesion sites compared to the cells on the planar reference. After 96 h, the MG-63 cells engulfed the micropillars and reached the bottom of the microtopography with their cell body indicating the before reported attempted phagocytosis. The interaction of integrins with their environmental cues activates the FAK by leading to its autophosphorylation of Y397. This phosphorylation of FAK provides binding sites for activated autophosphorylated Y419 Src and consequently the transphosphorylation of FAK Y861 by Src. Determination of the protein phosphorylation via luminex assay, which was then normalized on the total protein amount of FAK (Fig. 1b) and Src kinase (Fig. 1c), revealed no alteration for Src kinaseY419 phosphorylation but a significant decrease for the phosphorylation of FAK Y397 as well as FAK Y861 (Fig. 1b, c). The detected phosphorylation was normalized on the total FAK and Src protein amounts to exclude a possible different expression between the planar references and the micropillars. But beneath the different surface topography, the cells showed no significant changes in the expression of total FAK or Src protein.

The protein amount of paxillin, talin and vinculin as well as the phosphorylation state of paxillin were unchanged in MG-63 cells after 24 h on the micropillars (data not shown).

The development of focal adhesions and the regulation of actin stress fiber formation are stimulated by the small GTPase RhoA [17]. The major downstream regulator of RhoA is ROCK. ROCK-2 protein was decreased in MG-63 osteoblasts on the micropillars after 24 h (Fig. 1d). However, the amount of phosphorylated ROCK-2 relative to the amount of ROCK-2 protein was tendentially elevated for MG-63 cells on the micropillars (Fig. 1d).

### ROCK inhibition

**Fig. 2:**
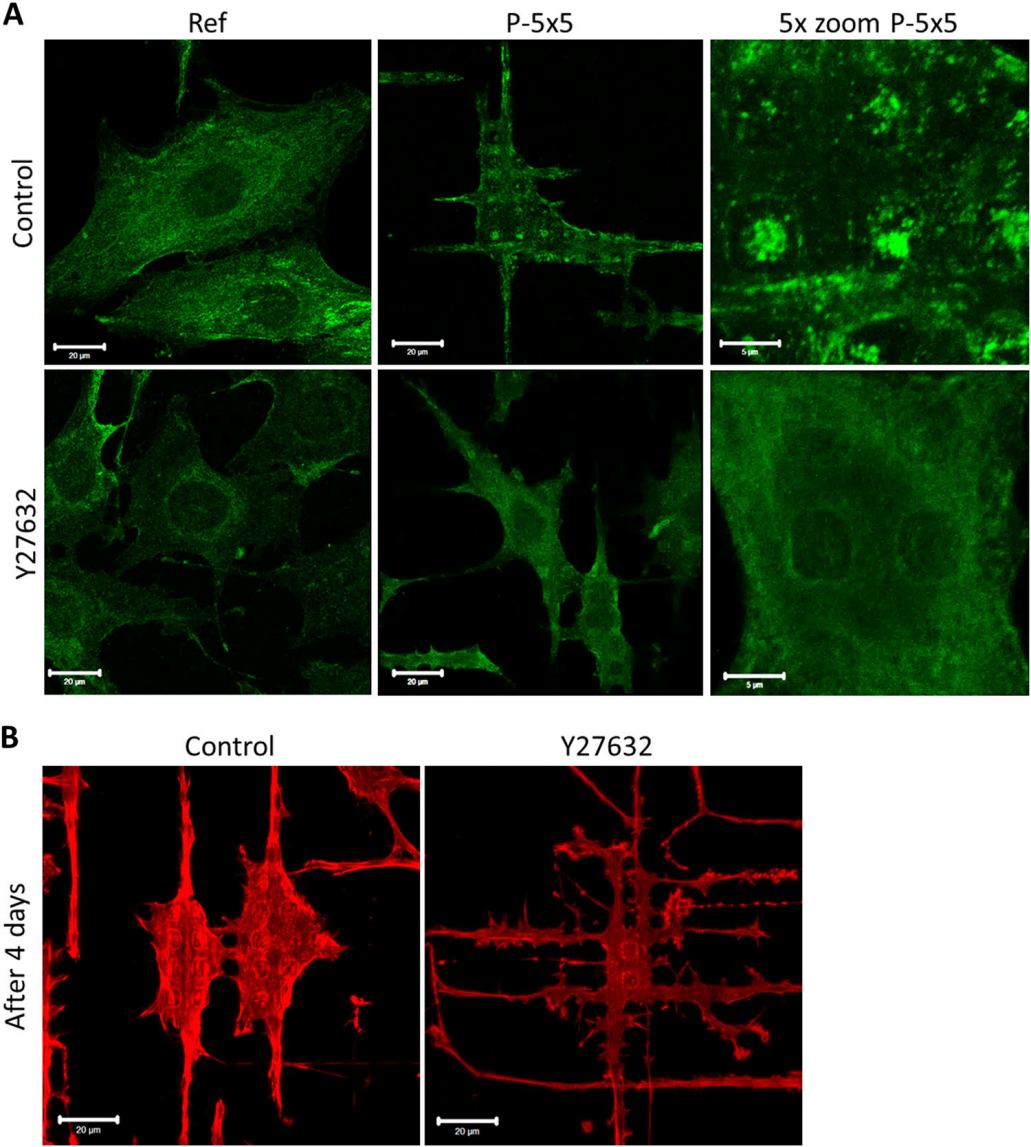
Immunofluorescence images of Cav-1 (green) and the actin cytoskeleton (red) of MG-63 osteoblasts grown on micropillars (P-5 × 5) and planar references (Ref). **a** Caveolin-1 (Cav-1) of 20 µM Y27632 treated cells for ROCK inhibition after 24 h. Note that the Cav-1 clustering on pillars was abrogated due to ROCK inhibition. **b** Actin cytoskeleton of 20 µM Y27632 treated cells for ROCK inhibition after 96 h on the micropillars P-5 × 5. Note the branched phenotype of cells after ROCK inhibition. (For (a) + (b): LSM780, Carl Zeiss, bars 20 µm and for 5x zoom 5 µm) (color figure online)

The integrin downstream signaling analysis showed alteration in the Rho/ROCK pathway for cells on the micropillars. Therefore, the cells were treated with the ROCK inhibitor Y27632. Rho/ROCK signaling is associated with alterations in the properties and signaling of membrane protrusions [18] and has a central role in many motile responses that involve the actin cytoskeleton, including phagocytosis [19]. Cells on micropillars were found to cluster the caveolin-1 (Cav-1) on top of the pillars (Fig. 2a), as also shown in [8]. Interestingly, after ROCK-2 inhibition with Y27632, the MG-63 cells on the micropillars exhibited no Cav-1 clusters on the micropillar plateaus (Fig. 2a). Following 96 h cultivation, the control osteoblasts concentrate their actin cytoskeleton on top of the pillars and, in addition are able to fully engulf the micropillars they lay on as seen by SEM [8]. In contrast, the Y27632 treated cells are more elongated and highly branched between the micropillar rows with their filopodia located at the bottom of the micropillars (Fig. 2b).

**Fig. 3:**
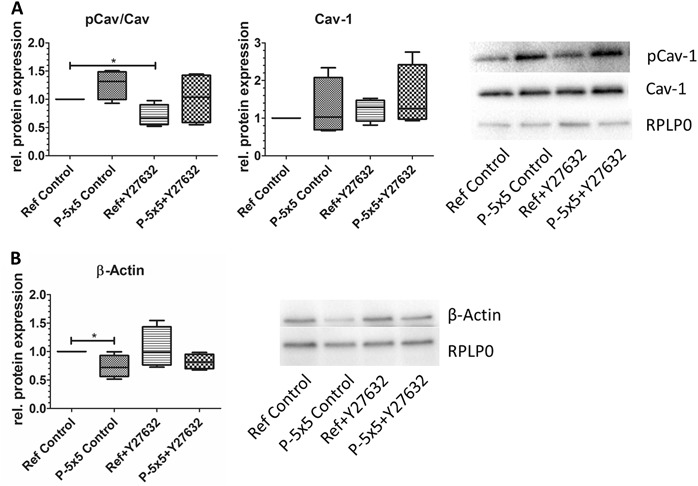
Influence of ROCK inhibition (20 µM Y27632) on protein expression of caveolin-1 (Cav-1) and β-actin in MG-63 osteoblasts on micropillars (P-5 × 5) and the planar references (Ref) for 24 h. **a** Phosphorylated caveolin (pCav-1) was calculated relative to Cav-1 (both normalized on the housekeeping RPLP0. **b** β-actin normalized on the housekeeping RPLP0. Note that ROCK inhibition is able to attenuate the pillar-induced decrease in β-actin. (For **a**+**b**: Ref values normalized on 1, *n* = 4, Mann-Whitney U test, *: *P* < 0.05)

The Cav-1 clustering on pillars was alleviated due to ROCK inhibition with Y27632. We wanted to know, if the protein expression level was also altered. Western blot analysis for the pCav-1 relative to the total Cav-1, showed a significantly decreased level after treatment with Y27632, but independent on the surface topography. This general lowering of the pCav/Cav levels results from a minor increase in Cav-1 protein expression after ROCK inhibition. After all, the micropillar-induced trend for an enhanced pCav/Cav was also observable after ROCK inhibition (Fig. 3a). The protein expression analysis for β-actin showed a significant reduction for the untreated MG-63 cells on the micropillars, which was not seen after ROCK inhibition with Y27632 (Fig. 3b).

### PI3K signaling and PIP2 phosphatidylinositols

**Fig. 4:**
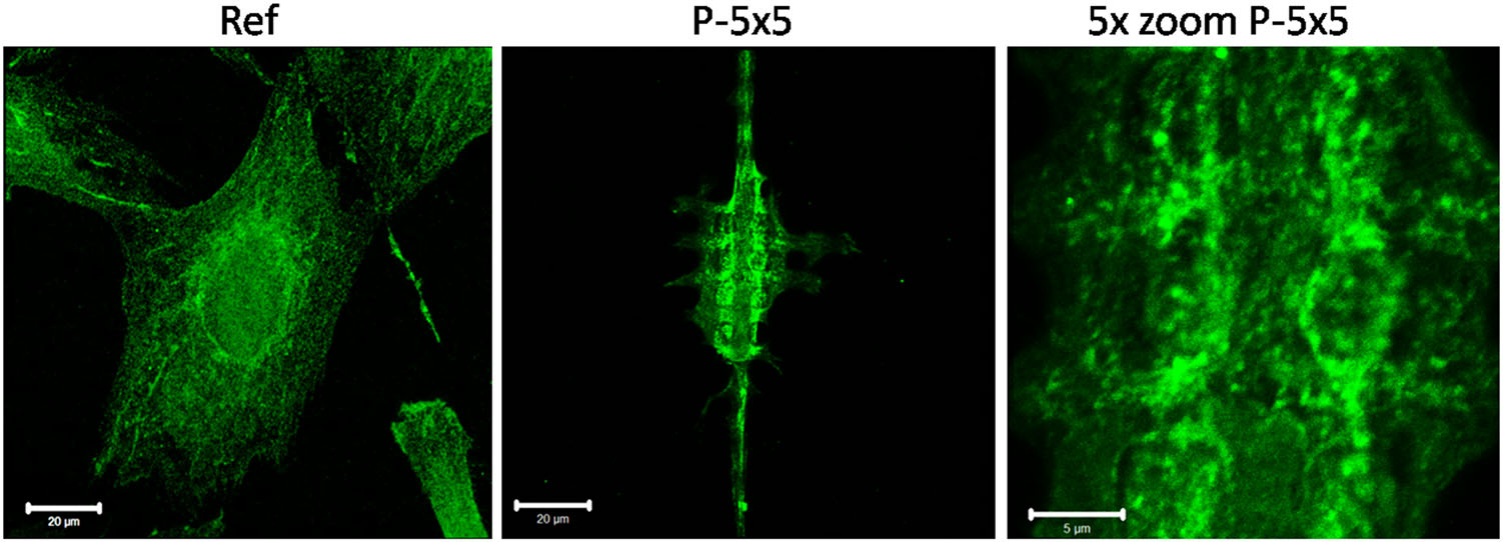
Immunofluorescence staining of the phosphatidylinositols in MG-63 osteoblasts grown for 24 h on micropillars (P-5 × 5) and the planar reference (Ref). PIP2 staining includes phosphatidylinositol-4-phosphate, phosphatidylinositol-4,5-bisphosphate (PI(4,5)P2) and phosphatidylinositol-3,4,5-trisphosphate (PI(3,4,5)P3). Note the clustered PIP2 around the micropillars. (LSM780, Carl Zeiss, bars 20 µm and for 5x zoom 5 µm)

The phosphoinositide 3-kinase (PI3K) signaling pathway plays a central role in the regulation of cell signaling, survival and proliferation. However, PI3K phosphorylation as well as the protein amount of PI3K showed no changes in MG-63 cells grown for 24 h on the micropillars (data not shown). In contrast, the phosphatidylinositol lipids, including phosphatidylinositol-4-phosphate, phosphatidylinositol-4,5-bisphosphate (PI(4,5)P_2_) and phosphatidylinositol-3,4,5-trisphosphate (PI(3,4,5)P_3_), displayed a changed localization in the cell membrane of MG-63 cells on the micropillars towards the top and around the micropillars after 24 h (Fig. 4). These phosphorylated inositols play an important role in the regulation of the cell movement.

**Fig. 5:**
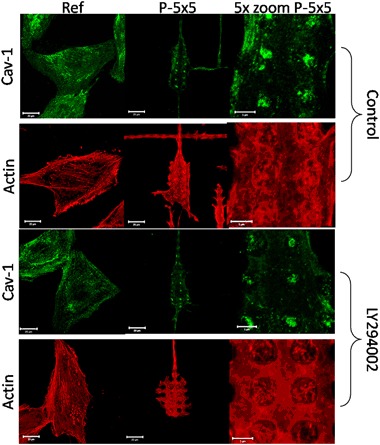
Caveolin-1 (Cav-1) and actin cytoskeleton organization in MG-63 osteoblasts after phosphatidylinositol 3-kinase (PI3K) inhibition with LY294002 (10 µM) after 24 h on micropillars (P-5 × 5). Note that inhibition of PI3K did not alter the caveolae membrane domain component Cav-1 and the actin cytoskeleton localization of the cells on the micropillars. (LSM780, Carl Zeiss, bars 20 µm and for 5x zoom 5 µm)

To see the PI3K influence on the organization of cellular structures more in detail PI3K inhibition with LY294002 was performed. But we revealed no alterations in MG-63 cells on micropillars concerning the intracellular actin cytoskeleton and the caveolae performance after 24 h (Fig. 5).

## Discussion

Osteoblasts are attempting to internalize the micropillar surface structures via a caveolae-mediated phagocytosis, to possibly establish the highest cell-surface contact to maintain their osteoblast function [8, 10]. The recognition and translation of the underlying topography information by the cells required complex signaling processing, starting with the integrins and the focal adhesions including their down-stream pathways. Focal adhesions were separated in (i) the integrin signaling layer containing integrin cytoplasmic tails, focal adhesion kinase (FAK), Src kinase and paxillin, (ii) an intermediate force-transducing layer with cytoskeletal adapter proteins such as talin and vinculin and (iii) an actin-regulatory layer.

In our experiments with osteoblasts on micropillars (24 h) the following scaffolding and signaling molecules were expressed in a stable, unchanged manner: the protein amounts of paxillin, talin, vinculin, PI3K, Rac, RhoA as well as the phosphorylation states of paxillin, PI3K, extracellular-regulated kinase (pERK1/2/ERK1/2), and mitogen-activated protein kinase (p-p38 MAPK/p38 MAPK) (data not shown).

However, the micropillar topography provides the MG-63 cells a reduced adhesion area; therefore they probably display less integrin-mediated autophosphorylation of FAK at its Y397. Because fewer binding sites at the FAK are accessible for the Src kinase, this results in the reduction of the FAK Y861 phosphorylation [18, 20]. Furthermore, the Src kinase showed no alternation in its phosphorylation state. This led to the suggestion that the Src kinase is also implicated in other processes, with the exception of the focal adhesion signaling for the MG-63 osteoblasts grown on micropillars. Src-family kinases are not only needed for the turnover of focal adhesions, but also for caveolin-1 (Cav-1) phosphorylation and internalization processes [17, 21]. Cav-1 can be phosphorylated at its Tyrosin14 by Src kinases. The Tyrosin14 phosphorylation of Cav-1 (pCav-1) is required for the recruitment of Cav-1 to the focal adhesions, where Cav-1 associates with β1-integrins and links the integrins to Src family kinases [21]. The phosphorylation of Cav-1 was reported to be enhanced in MG-63 cells on the micropillars [8]. Possibly because of the increased Cav-1 phosphorylation by the Src kinase, Src kinase phosphorylation showed no alteration for the osteoblasts on the micropillars. The early phase of adhesion involves integrin-mediated activation of Rac and Cdc42 for actin polymerization for the cell spreading, and in the later phase RhoA activation, which lead to increased contractility as well as tension transmission. After 24 h observation time, the early phase could be passed and in the later phase of adhesion, the Ras homolog family member A (RhoA)/ROCK pathway plays the essential role for the actin cytoskeleton regulation including many motile processes such as phagocytosis. ROCK mediates the RhoA-induced actin bundle formation and regulates focal adhesion maturation [19]. The isoform ROCK-1 has a major role in the actin stress fiber formation, whereas ROCK-2 is rather involved in processes that require phosphoinositide 3-kinase (PI3K) and myosin activity such as phagocytosis [22].

Consequently, the cells showed signaling enhanced for motile processes, such as the reported caveolae-mediated phagocytosis accompanied with an increased Cav-1 phosphorylation (pCav-1) [8]. The pCav-1 was reported to stimulate Rho activation and ROCK interaction in an Src-dependent manner, resulting in stabilized FAK association within the focal adhesions and promoted cell migration [23]. Thus, the slightly elevated Src-phosphorylation (as shown in Fig. 1c) and Cav-1 phosphorylation [8] in MG-63 cells grown for 24 h on the micropillars likely lead to the ROCK activation. In consequence, this ROCK signaling transduces the phagocytosis signals for the RhoA-dependent actin organization.

Phosphatidylinositols also play a central role in endocytic processes, such as the reported caveolae-mediated phagocytosis [10]. These phosphorylated inositols play an important role in the regulation of cell movement [15] and in indicating the areas for the PI3K activity, which uses PI(4,5)P_2_ as a substrate and converts it into PI(3,4,5)P_3_ [24]. During phagocytosis, phosphoinositides experience a sequential turnover in which PI(4,5)P_2_ is located in nascent endosomes and is converted by the PI3K to PI(3,4,5)P_3_ in the late engulfment phase. Thus, the PI3K is essential for the closure of the endocytic vesicle [18]. But on the micropillars, the cells never reaches the late engulfment phase in which the PI3K is essential, because the micropillars are fixed to the surface. Therefore, the micropillars cannot be fully internalized by the cells; therefore the PI3K inhibition has no impact on the cells grown on the micropillars. Diminished particle phagocytosis after PI3K inhibition as well as the essential role for PI3K in the engulfment of large targets was reported before [25].

After ROCK inhibition, the cells may establish the highest cell-surface contact while avoiding the caveolae-mediated micropillar phagocytosis by performing the high branched morphology. The ROCK inhibition did not alter Cav-1 phosphorylation, which elucidates the importance of ROCK in the Cav-1 activation for cluster formation and verifies that ROCK is an important component for the topography-sensing and processing of the osteoblasts. In the later phase of adhesion, RhoA/ROCK are activated for transmission of tension and integrin ligation [26], which are shown to be important as well for the caveolae-mediated surface internalization by osteoblasts. The key feature in environmental signaling is the crosstalk between the integrins and actin [27], presumably over the Rho/ROCK pathway concerning the surface topography. In addition, RhoA was reported to be important for topography-induced focal adhesion formation and FAK phosphorylation [25]. Signaling pathways, such as the Rho/ROCK pathway, which interconnect and feedback actin with integrin receptors, are essential for the sensory and function of focal adhesions [27] as well as the sensing of the topography and the transduction of the topography information.

## Conclusion

This study highlights the involvement of the intracellular signaling protein ROCK concerning the recognition of surface topographies by osteoblasts as well as in the transduction of the topography information in cellular responses. In detail, we could show that the inhibition of ROCK-2 with Y27632 impairs cellular processes such as phosphorylation of caveolin-1 and actin clustering around sharp-edged microtopographies, which are involved both in the attempt to phagocytize the micropillars. The phosphatidylinositol 3-kinase (PI3K) inhibition with LY294002 for 24 h did not influence the Cav-1 clustering on micropillars. The understanding of cellular signaling in more detail is important to find out specific cell parameters which react sensitive to topographic features of the biomaterial surface.

## Abbreviations

Cav-1: Caveolin-1
ECM: Extracellular matrix
FAK: Focal adhesion kinase
MFI: Mean fluorescence intensity
PI3K: Phosphatidylinositol 3-kinase
PI(4,5)P_2_: Phosphatidylinositol-4,5-bisphosphate
PI(3,4,5)P_3_: Phosphatidylinositol-3,4,5-trisphosphate
ROS: Reactive oxygen species
ROCK: Rho-associated kinase
RhoA: Ras homolog family member A
RPLP0: 60S acidic ribosomal protein P0
S: Serine
Ti: Titanium
Y: Tyrosine
